# Supplementary material for: Arsenic Removal via the Biomineralization of Iron-Oxidizing Bacteria Pseudarthrobacter sp. Fe7
Source: Microorganisms. 2023 Nov 26;11(12):2860. doi: 10.3390/microorganisms11122860 (PMC10746119; doi:10.3390/microorganisms11122860)
Supplement: Supplementary file 1 [file microorganisms-11-02860-s001.zip › microorganisms-2722983-supplementary.pdf]

Arsenic Removal via the Biomineralization of Iron-Oxidizing Bacteria *Pseudarthrobacter* sp. Fe7

Xia Fan [1], Hanxiao Zhang [1], Qian Peng[1], Yongliang Zheng [1], Kaixiang Shi [2]* and Xian Xia [3]*

[1] College of Biology and Agriculture Resources, Huanggang Normal University, Huanggang 438000, China; fanxia@hgnu.edu.cn (X.F.); 15038881601@163.com (H.Z.); m18040651471@163.com (Q.P.); zylgolden@126.com (Y.Z.)
[2] National Key Laboratory of Agricultural Microbiology, College of Life Science and Technology, Huazhong Agricultural University, Wuhan 430070, China
[3] Hubei Key Laboratory of Edible Wild Plants Conservation & Utilization, Hubei Engineering Research Center of Characteristic Wild Vegetable Breeding and Comprehensive Utilization Technology, College of Life Science, Hubei Normal University, Huangshi 43500, China
* Correspondence: kaixiangshi@mail.hzau.edu.cn (K.S.); xianxia@hbnu.edu.cn (X.X.)

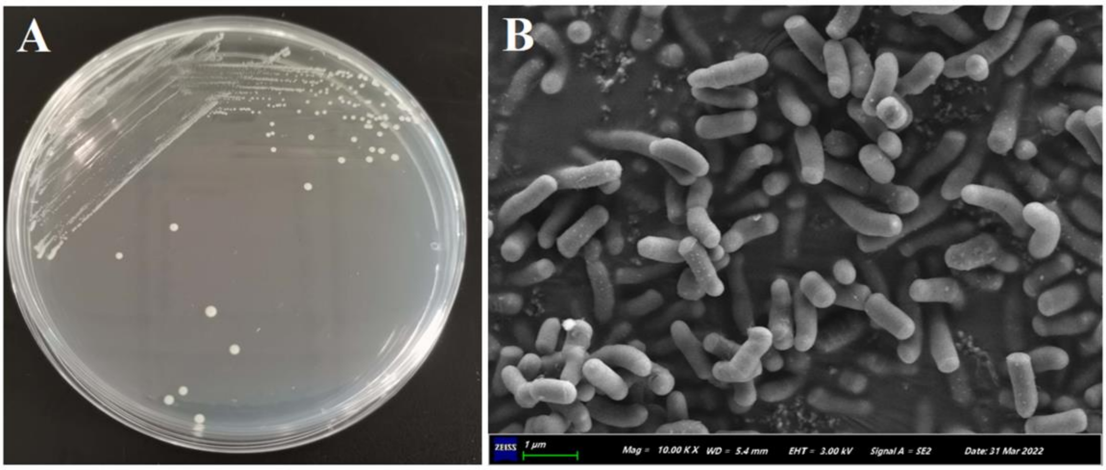

Figure S1. (A) Colony morphology and (B) SEM image of strain Fe7.

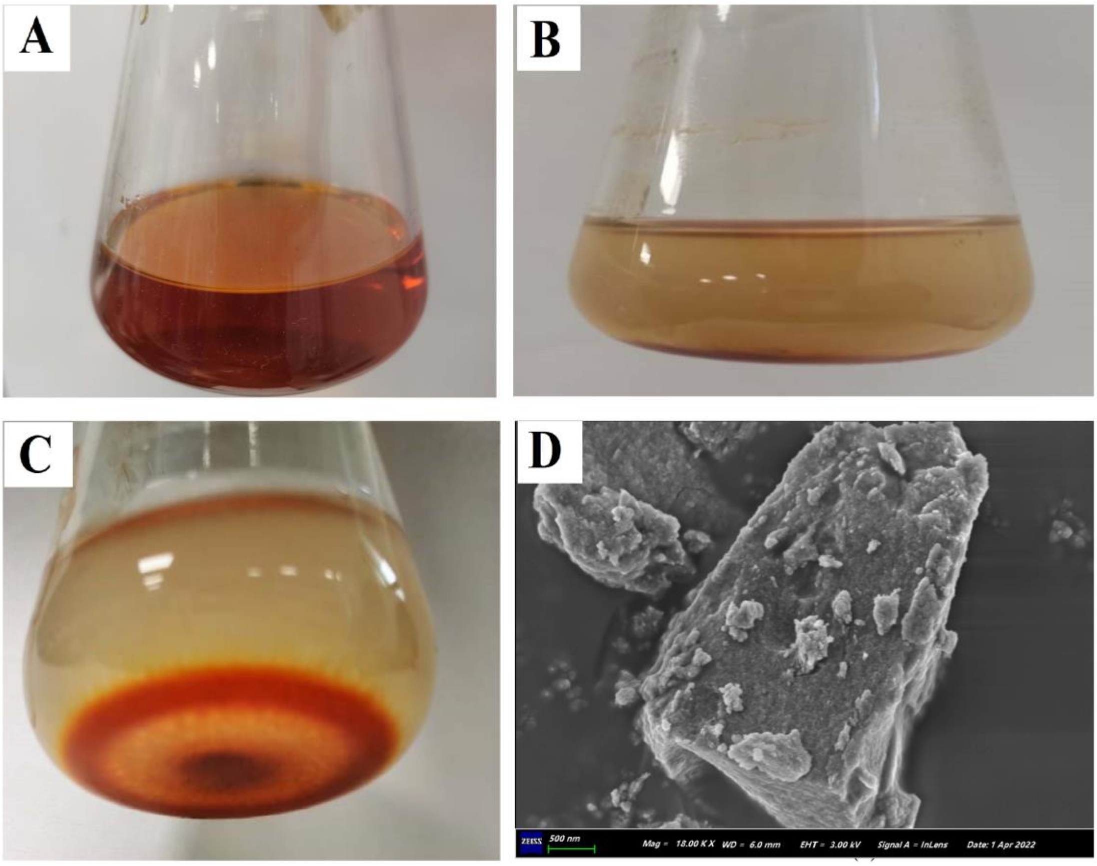

Figure S2. Growth status of strain Fe7 in a modified PYCM liquid medium and SEM image of the iron precipitate produced by strain Fe7. (A) Medium without strain Fe7. (B) Cell suspension. (C) Biological iron precipitates. (D) SEM pattern.
